# Solid-State Form Characterization of Riparin I

**DOI:** 10.3390/molecules22101615

**Published:** 2017-10-09

**Authors:** Elisana Afonso de Moura, Márcio Vinícius Cahino Terto, Elisângela Afonso de Moura Mendonça, José Valdilânio Virgulino Procópio, Vicente Carlos de O. Costa, José Maria Barbosa Filho, Stanley Juan Chavez Gutierrez, Josean Fechine Tavares, Rui Oliveira Macedo, Marcelo Sobral da Silva

**Affiliations:** 1 Pharmaceutical Sciences Departament, Federal University of Paraíba, University City, João Pessoa PB 58059-970, Brazil; viniciuscahino@hotmail.com (M.V.C.T.); jose.procopio@ltf.ufpb.br (J.V.V.P.); vicente@ltf.ufpb.br (V.C.d.O.C.); jbarbosa@ltf.ufpb.br (J.M.B.F.); josean@ltf.ufpb.br (J.F.T.); ruimacedo@ccs.ufpb.br (R.O.M.); marcelosobral.ufpb@gmail.com (M.S.d.S.); 2 Biotechnology Departament, Federal University of Paraíba, University City, João Pessoa PB 58059-970, Brazil; elisdoc@gmail.com; 3 Pharmaceutical Sciences Departament, Federal University of Piauí, Teresina PI 64600-000, Brazil; stanley@ltf.ufpb.br

**Keywords:** riparin I, solid-state characterization, pharmaceutical analysis

## Abstract

Riparin I is an alkamide with potential anxiolytic activity in preclinical studies. The characterization and understanding of solid-state properties play an importance role in drug development. For this work, the solid state of five riparin I batches (RIP-1, RIP-2, RIP-3, RIP-4, and RIP-5), obtained by the same synthesis process, were characterized by Scanning Electron Microscopy (SEM), Differential Scanning Calorimetry (DSC), DSC-photovisual, Thermogravimetry (TG), Fourier Transform Infrared (FTIR), Pyrolysis (Pyr-GC/MS), X-ray Powder Diffraction (PXRD), and Solid-State Nuclear Magnetic Resonance (ssNMR) techniques. Batches of riparin I with different crystal habits resulting in crystallization impurities were observed, which can be attributed to the presence of triethylamine. The main differences were observed by DSC, PXRD, and ssNMR analysis. DSC curves of RIP-2 and RIP-3 presented endothermic peaks at different temperatures of fusion, which can be attributed to the mixture of different crystalline forms. PXRD and ssNMR results confirmed crystallinity differences. The results offer evidence of the importance of controlling the reproducibility of the synthesis in order to obtain the adequate morphology for therapeutic efficacy and avoiding future problems in quality control of riparin I products.

## 1. Introduction

The physical characterization of pharmaceutical solids plays an important role in the initial stages of the development of new drugs. Since solid-state active pharmaceutical ingredients (APIs) can have different crystalline structures, polymorphism is a major concern for the pharmaceutical industry, due to its implications for physicochemical and biopharmaceutical properties [1].

The diversity of solid-state forms that drugs may attain is typically governed by different factors such as the synthetic route and reaction conditions; the quality of the starting materials, reagents, and solvents; and the purification steps for the end product [2,3]. Batch-to-batch variations of the concentration of impurities during the crystallization of organic products are known to lead to significant changes in the quality of the final crystal [4,5].

The reproducible behavior of drug formulations may depend critically on the precise solid form of the drug employed and the isolation and thorough characterization of the various forms. Therefore, quality control of the APIs is necessary in order to ensure the consistent batch-to-batch quality of pharmaceutical products [6,7].

Understanding the differences in the physical properties of the solid forms and their relative stabilities is, therefore, essential for pharmaceutical manufacturers in relation to the selection of a particular form that demonstrates the desired characteristics for achieving maximum therapeutic efficacy in the initial stages of drug development [8].

Various analytical methods have been employed to characterize and monitor the solid forms of drugs during the various steps of processing and development, including powder X-ray diffraction (PXRD), infrared spectroscopy, scanning electron microscopy (SEM), thermal analysis, and solid-state nuclear magnetic resonance (ssNMR). Traditionally, the study of solid-form diversity has needed to rely on the use of a combination of techniques to avoid errors in interpretation and in the identification of crystalline forms [9,10,11,12].

Powder X-ray diffraction analysis is an indispensable tool for the characterization and quality control of crystalline and amorphous materials. PXRD is widely employed and produces a diffraction pattern that can be used as a type of fingerprint; thus, it can be utilized to screen polymorphs during drug discovery, formulation development, and manufacturing [11]. Pyrolysis and thermal analysis techniques include differential scanning calorimetry (DSC) and thermogravimetric analysis (TGA); these thermal methods are commonly used to determine the characterization and thermal decomposition of drugs and in the evaluation of their stability [13].

**Figure 1:**
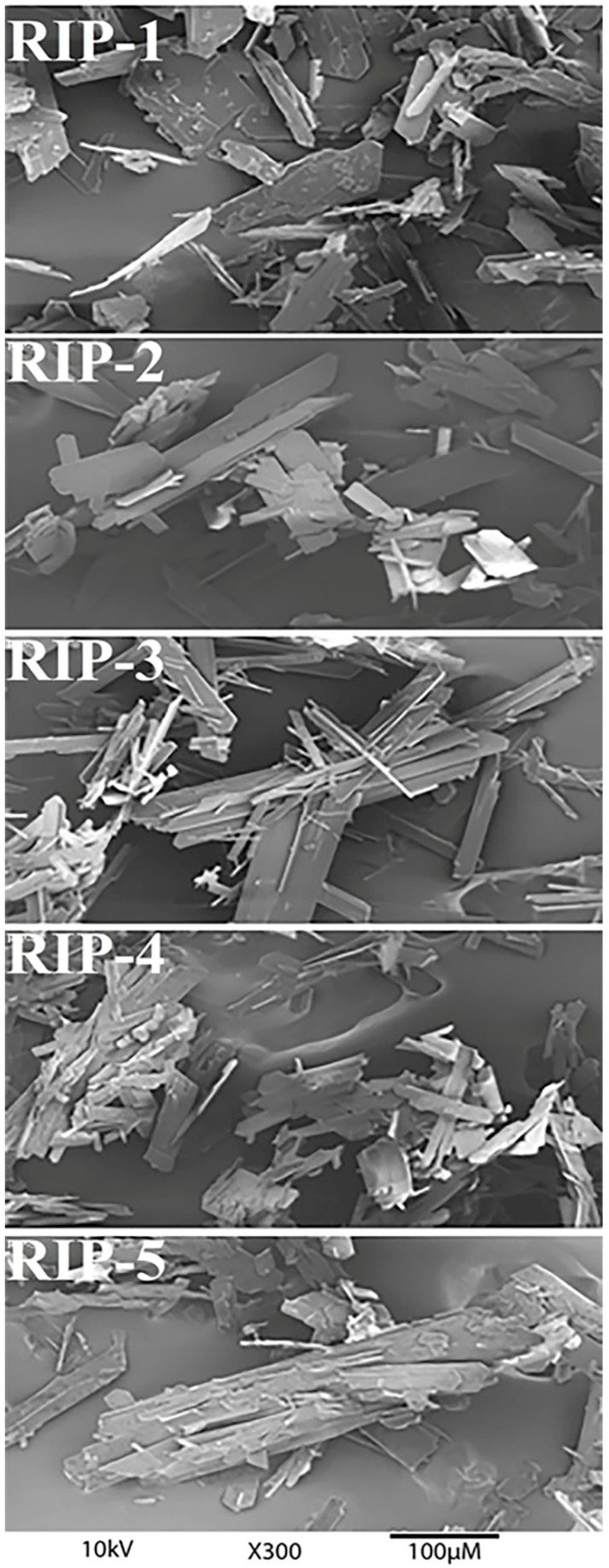
Scanning electron microscopic photographs of riparin I batches.

The methyl ether of *N*-benzoyl tyramine is called riparin I (Figure 1) and is an alkamide isolated from the green fruit of *Aniba riparia* or synthesized by Barbosa Filho et al. [14]. In Brazil, its biological properties have been intensively investigated. When administered orally or intraperitoneally in mice, it shows anxiolytic effects yet without any sedative or muscle-relaxing effects, thereby eliminating the common side effects associated with classic benzodiazepines [14,15,16]. In spite of the pharmacological importance of riparin I, characterization of the solid-state form of riparin I obtained by the synthesis process in order to produce the solid form that is of interest has not been reported in the literature.

For this reason, the objective of the present work was to characterize and compare five batches of riparin I produced by the same synthetic process. The aim was to evaluate their quality by means of solid-state characteristics, employing scanning electron microscopy, differential scanning calorimetry, thermogravimetric analysis, infrared spectroscopy, powder X-ray diffraction, pyrolysis coupled with mass spectrometry (Pyr-GC/MS), and solid-state nuclear magnetic resonance.

## 2. Results and Discussion

### 2.1. Scanning Electron Microscopy (SEM)

SEM analysis revealed riparin I batches with particles presenting distinct morphologies and crystalline habits (Figure 1). The photomicrographs revealed that the non-homogeneous particle size of the samples exhibited an irregular surface with the appearance of non-ordered plates and a tendency to agglomerate. The RIP-1 batch exhibited plates with wide edges, with smaller particle fragments on the surface. The RIP-2 exhibited plates with more tapered edges without smaller particle fragments on the surface. The RIP-3 exhibited plates with more tapered edges, and fragments of crystals with well-defined acicular habits were deposited on the surface. The RIP-4 exhibited uniform plate-like crystals and small-sized particles. The RIP-5 showed multi-layered plates that overlapped. The results indicated that batches displayed obvious differences in morphology due to the different molecular arrangements of solid-state riparin I. These differences in particle size and morphology can be caused by polymorphism or by changes in the crystal growth conditions, the nature of the crystallization solvent, the degree of supersaturation, or the presence of impurities [2]. Differences in the crystal habit may strongly influence particle orientation and modify the flowability, packing, compressibility, and dissolution characteristics [1].

### 2.2. Differential Scanning Calorimetry (DSC)

**Figure 2:**
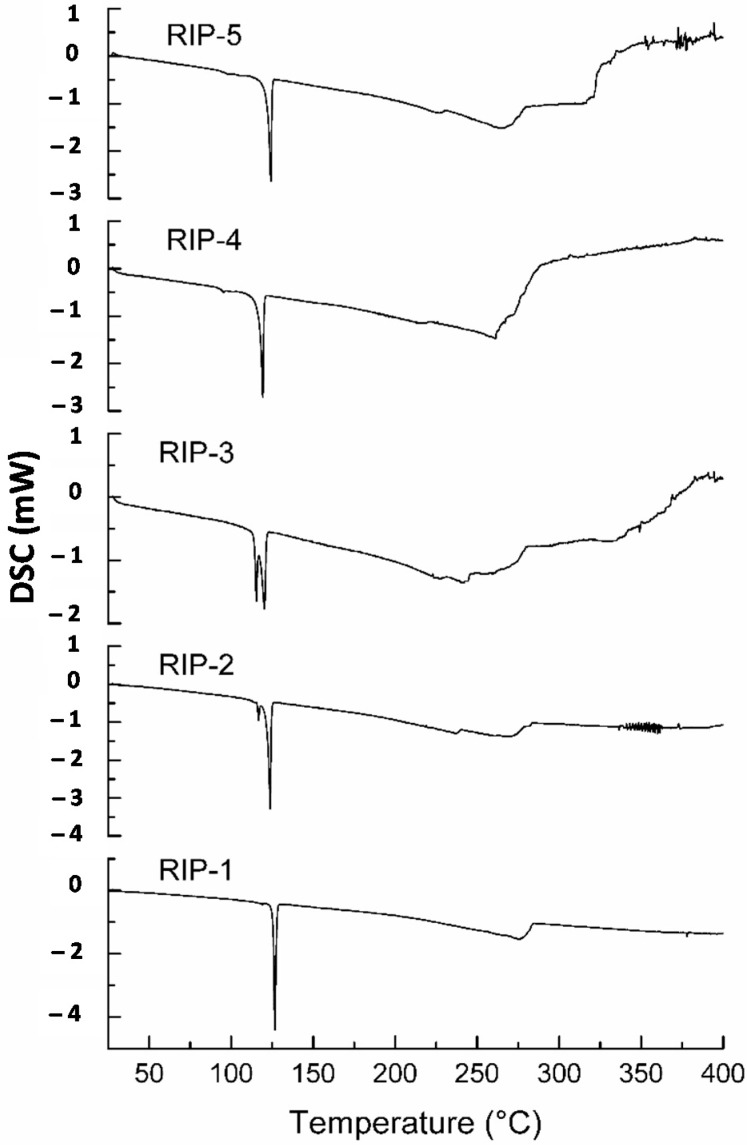
DSC curves of riparin I batches at the heating rate of 2.0 °C·min^−1^.

The DSC curves of riparin I batches were obtained at a heating rate of 2.0 °C min^−1^ (Figure 2). The calorimetric curve of the RIP-1 batch showed a single sharp endothermic peak event at 125.7 °C (ΔH = −100.6 J·g^−1^) corresponding to the melting point, suggesting an anhydrous form. By contrast, the calorimetric curves of RIP-2 and RIP-3 revealed the existence of two endothermic events. The first endothermic event for RIP-2 and RIP-3 occurred at temperatures of 118.7 °C (ΔH = −6.1 J·g^−1^) and 118.2 °C (ΔH = −15.8 J·g^−1^), respectively. The second endothermic event occurred at 125.2 °C (ΔH = −80.9 J·g^−1^) for RIP-2 and 123.9 °C (ΔH = −30.2 J·g^−1^) for RIP-3. This phenomenon suggests the solvent molecules’ crystal lattice or the occurrence of different crystalline structures [7]. DSC curves for RIP-4 and RIP-5 batches showed a single endothermic peak at 123.7 °C (ΔH = −73.5 J g^−1^) for RIP-4 and 124.4 °C (ΔH = −69.3 J·g^−1^) for RIP-5, suggesting an anhydrous form. Despite similar thermal behavior in RIP-1, RIP-4, and RIP-5 batches, the DSC data showed variations in temperatures and heat fusion, suggesting differences in crystallinity. The DSC data observed for batches of riparin I present different thermal behaviors, confirming the different solid-state forms in agreement with SEM and suggesting the mixture of forms of itself.

### 2.3. DSC Coupled with the System Photovisual

**Figure 3:**
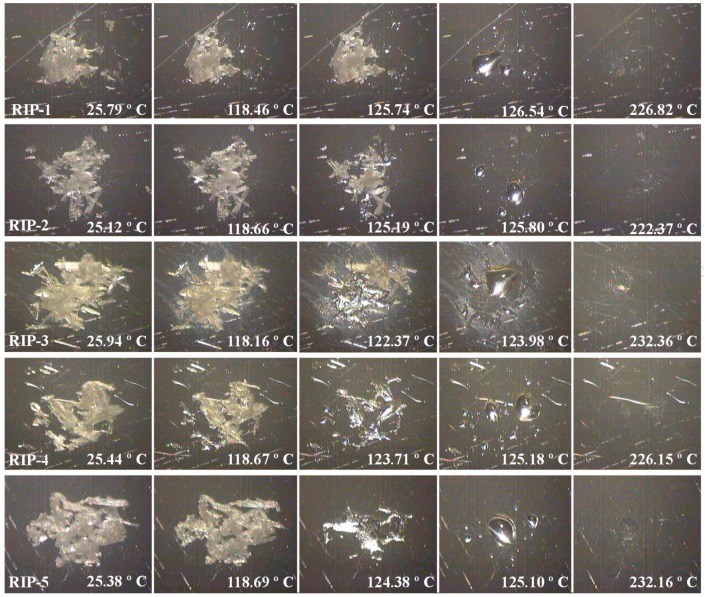
DSC-Photovisual of RIP-1, RIP-2, RIP-3, RIP-4, and RIP-5 samples in the heating 400 rate of 10 °C·min^−1^.

In the images obtained in the DSC-Photovisual shown in Figure 3, substantial differences could be observed among the riparin I batches, confirming the DSC convectional data. For the RIP-1 batch, it is possible to observe the melting point at a temperature range of 125.7–126.4 °C; this occurred in a uniform process manner and was well defined in all the sample extensions. For the RIP-2 batch, in the image obtained at 124.3 °C, the initial melting process was observed, corresponding to the phase transition at a temperature range of 118.0–125.3 °C visualized in the convectional DSC curve. The image obtained for RIP-3 at a temperature of 122.4 °C showed two events corresponding to the melting process of the crystalline form as less stable, and the form more stable staying unaltered. The following image, at a temperature of 123.9 °C, observed the melting process of the crystalline form as more stable. The images confirm the presence of different crystalline forms visualized in the conventional DSC curves. The images corresponding to the melting points for RIP-4 and RIP-5 showed the process only at the temperature ranges of 123.7–125.1 °C and 124.3–125.1 °C, respectively, with the phase transition for both evolved to low enthalpy. The images at the temperatures of 226.8 °C, 222.37 °C, 232.26 °C, 226.15 °C, and 232.16 °C, for RIP-1, RIP-2, RIP-3, RIP-4, and RIP-5, respectively, correspond to a volatilization of the samples studied.

### 2.4. DSC Coupled with the System Photovisual

**Figure 4:**
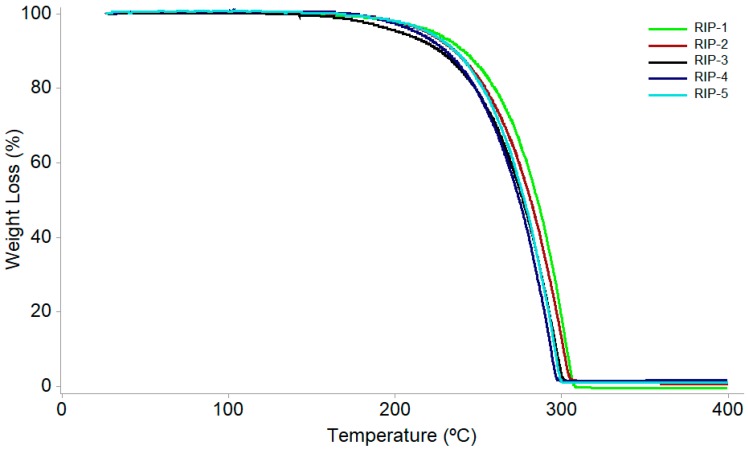
Dynamic thermogravimetric curves of riparin batches at the heating rate of 10.0 °C·min^−1^.

The thermogravimetry curves present similar thermal behavior for RIP batches 1–5, in regard to the thermal decomposition process taking place in a single stage in all the samples and the studied heating rates. The RIP-1 batch shows mass loss in the temperature range of 249.1–304.6 °C, with the percentage of mass loss at 99.4%; RIP-2 in the range of 245.4–299.37 °C with 99.8% of mass loss; RIP-3 in the range of 168.4–300.2 °C with 98.8% mass loss, RIP-4 in the range of 218.1–296.4 °C with a mass loss of 99.8%, and RIP-5 in the range of 222.7–302.2 °C and a mass loss of 99.8% (Figure 4). Thus, it is possible to infer that the samples have different thermal stability, indicating a thermal stability order in which batch RIP-3 < RIP-4 < RIP-2 < RIP-5 < RIP-1. Ozawa’s kinetic method, obtained from the dynamic TG data, has been employed to evaluate the kinetic parameters of order-reaction (*n*), activation energy (Ea), and frequency factor (A) using the decomposed fraction α10 between the start and end of the step decomposition (Table 1). The data suggest a thermal process with a kinetic of zero order for all batches of riparin I [17,18]. Additionally, it was not possible to observe a significant mass loss in the temperature range of 30.2–150.0 °C for the phase transitions observed in the DSC-50 for all batches in the TG curve. RIP-1, RIP-2, RIP-3, RIP-4, and RIP-5 presented 0.18, 0.03, 0.08, 0.03, and 0.12% mass loss, respectively, demonstrating the absence of solvents in the crystal structure, which implies that the analyzed crystals are not solvated or hydrated forms [16].

### 2.5. Fourier Transform Infrared (FTIR)

**Figure 5:**
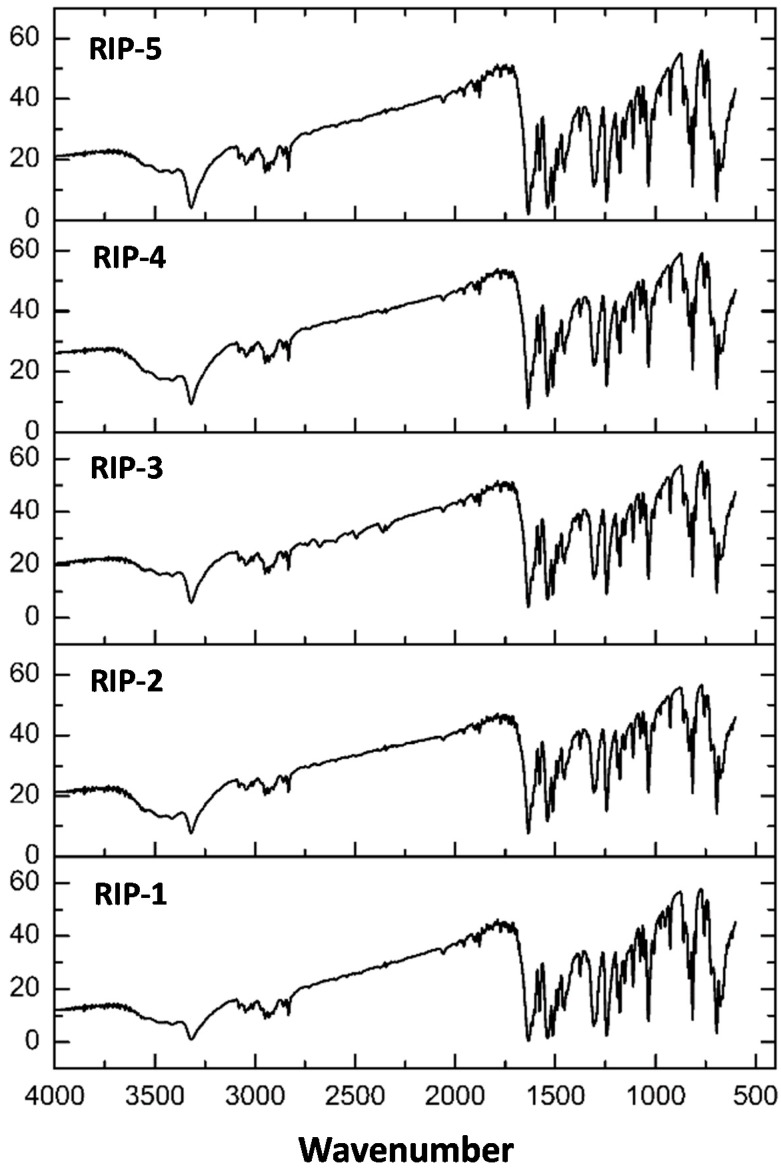
FTIR spectra of riparin batches.

Through the infrared vibrational spectroscopy analysis, responses can be obtained based on the vibration of specific molecules, in which it is possible to characterize hydrates, solvates, and different crystalline forms [7]. In Figure 5, the infrared spectra obtained for samples RIP-1–5 can be seen. The characteristic absorption bands of riparin I in the IR spectrum are N-H at 3325 cm^−1^, C=O in 1637 cm^−1^, and stretch C=C of aromatic in 1535 and 1512 cm^−1^ [19].

The results of the spectra confirmed the presence of the characteristic functional groups of the riparin I molecule for all batches, in addition to chemical shifts for these groups (Table 2). The obtained data corroborate the results of TG curves, allowing us to determine the absence of solvents in the crystalline structure of the five batches of riparin I, which infers that these are anhydrous crystalline forms [7]. The largest difference observed in the IR spectra of the batches RIP-2 and RIP-3 is the shift of the band 3325.49 to 3323.56, which is related to the N-H. However, it is difficult to determine if these differences are caused by distinct crystalline forms.

### 2.6. Pyrolysis Coupled with Gas Chromatography/Mass Spectrometry (Pyr-GC-MS)

**Figure 6:**
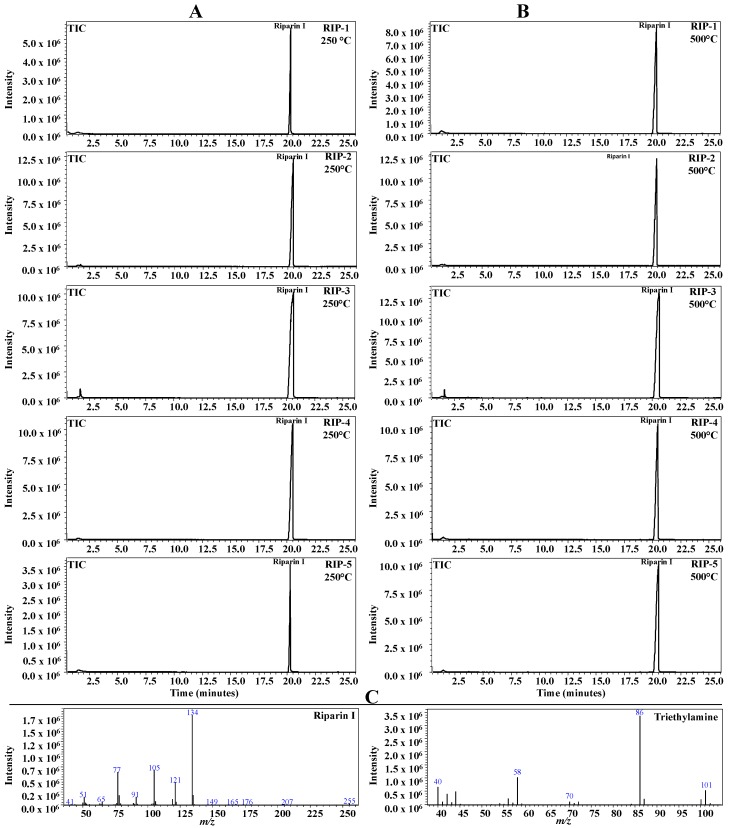
Pyrograms obtained from riparin at the temperatures of 250.0 °C (column (**A**)) and 500.0 °C (column (**B**)), showing the peak corresponding to riparin I and the product ion mass spectra of riparin I and triethylamine (column (**C**)—bottom of the figure).

The degradation products corresponding to the only step of riparin I mass loss in the dynamic TG were studied by pyrolysis coupled with GC-MS. The pyrograms obtained at temperatures of 250 °C and 500 °C are shown in Figure 6. The molecular ion of riparin I in the ratio of mass:charge (*m*/*z* 255.0) was identified in the spectra of all batches. The data suggest that the mass loss observed on the TG (Figure 4) does not correspond to the decomposed products of the drug, but to the volatilization of the riparin in its intact molecular form. The data confirm zero order of decomposition [17,18]. Due to the technique’s sensitivity [20], a peak detected at the beginning of the pyrogram, identified as triethylamine for all batches, was later found to be an impurity in the synthesis process [21].

### 2.7. Powder X-Ray Diffractometry (PXRD)

**Figure 7:**
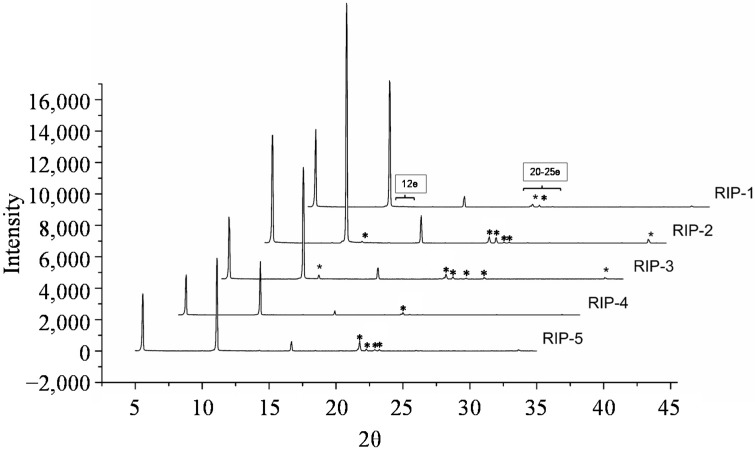
PXRD patterns of riparin I batches. The (*) show characteristic diffraction pattern for each lot.

The diffraction patterns for different batches of riparin I RIP1–5 have sharp peaks, showing batches with crystallinity characteristics and the absence of amorphization (Figure 7). The PXRD patterns for batches exhibited three characteristic peaks with diffraction of significant intensity at 5.5°, 11.1° and 16.6° (2θ) for all batches. However, crystallographic differences were observed by the presence of distinct peaks at the angle 12° only for RIP-2 and RIP-3 batches, which showed two characteristic endothermic melting events in a conventional DSC. Additionally, it is possible to observe PXRD patterns in the range of 20° to 25°, distinct for all batches. A peak was also noted at 34°, which is characteristic of the C=O group out of the plane from the closest benzene ring [13]. The variations of the intensities of the peaks can be attributed to the differences related to the size of the crystallite [22]. These observations confirm the information obtained in the thermal data and SEM, DSC, and TG, providing evidence of the differences in the characteristics of the solid-state crystal habit of different batches.

### 2.8. Solid-State Nuclear Magnetic Resonance (ssNMR)

**Figure 8:**
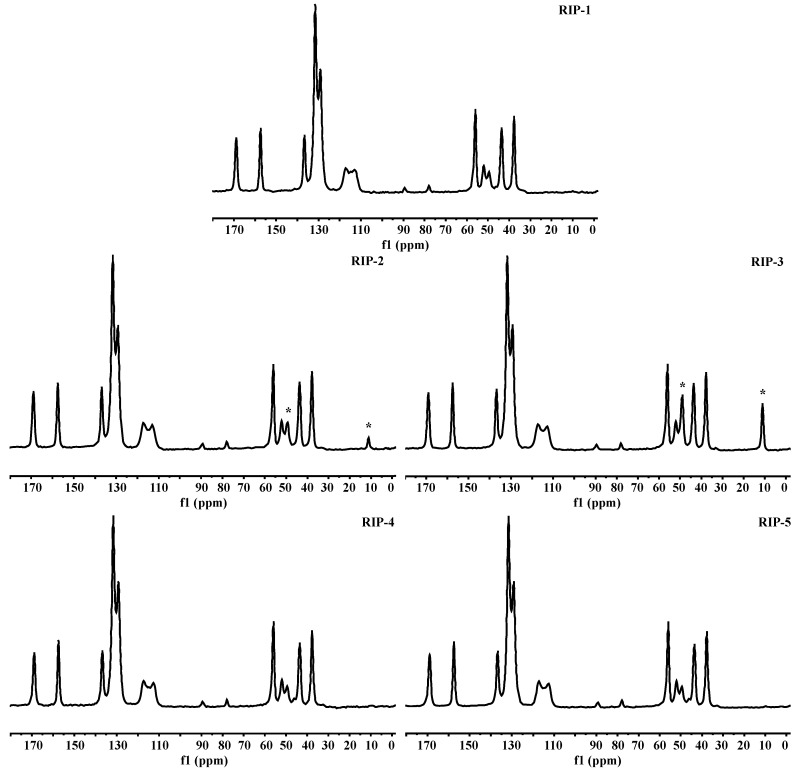
Solid-state ^13^C-NMR spectra of riparin I physical forms. The (*) show characteristic peak corresponding of triethylamine.

The riparin I batches were also studied by ssNMR. The ^13^C-NMR spectra of batches are shown in Figure 8. The solid forms can be clearly distinguished based on their high-resolution ssNMR spectra [1,2]. The data show a well-resolved signal, indicating that there is only one molecule in the crystalline asymmetric for RIP-1, RIP-4, and RIP-5. However, differences shown in the ssNMR spectra for batches RIP-2 and RIP-3 indicated the presence of new peaks with a signal at 11.18 ppm, corresponding to the methyl group, confirming the compound triethylamine as an impurity, detected by Pyr-GC-MS. Thus it can be confirmed that the impurity caused the crystal lattice to change the local chemical environment and caused the observed NMR change. The triethylamine presence can be attributed to poor control of the synthetic process steps, resulting in the starting materials being incorporated into the crystal lattice [5]. The safety of a drug product is dependent not only on the toxicological properties of the API itself but also on the impurities that it may contain. Moreover, this problem can influence crystal nucleation and growth during this process, resulting in batch-to-batch variations in the quality of the final solid product [3,10,21]. The data show the relevance of the optimization of the operating conditions for manufacturing the drug, while these parameters are of tremendous importance to determine the quality of the product.

## 3. Materials and Methods

### 3.1. Materials

The riparin I batches were prepared by a collaborator of our research group. Five batches, named RIP-1, RIP-2, RIP-3, RIP-4 and RIP-5, were evaluated. All chemicals used were of pharmaceutical analytical grade.

### 3.2. Scanning Electron Microscopy (SEM)

The photomicrographs were taken at a voltage of 10 kV and a magnification of 300×, 500× and 1000× using a Jeol JSM 6060 microscope (Joel Electron Microscope, Tokyo, Japan). The batches were fixed on brass stubs using double-sided adhesive tape and vacuum-coated with a thin layer of gold.

### 3.3. Differential Scanning Calorimetry (DSC)

The DSC curves of riparin I batch were recorded in triplicate using a differential scanning calorimeter from Shimadzu (model DSC-50, Tokyo, Japan). The apparatus was calibrated with indium (153.3 ± 0.3 °C) as standard. The heat flow signal was calibrated to the melting heat of indium (28.59 ± 0.3 J·g^−1^). DSC curves were recorded at a heating rate of 2.0, 5.0, 10.0, 20.0 and 40.0 °C·min^−1^, in the temperature range 25–400 °C, in dry nitrogen flow 50.0 mL·min^−1^. The samples (2.0 ± 0.2 mg) were packed in an aluminum crucible. The crucible was sealed hermetically. The DSC data were analyzed using Tasys^®^ software (TA-60WS) from Shimadzu.

### 3.4. DSC Coupled with a Photovisual System (DSC-Photovisual)

The DSC photovisual data were recorded with a differential scanning calorimeter (Shimadzu, model DSC-50) coupled with a photovisual system, equipped with a microscope (Olympus, Tokyo, Japan, model SZ-CTV60) and a camera (Sony, Tokyo, Japan, model VCC-520). The batches were placed into an aluminum crucible and heated in the temperature range 25–400 °C at a heating rate of 10 °C·min^−1^ under the same conditions of nitrogen flow from conventional DSC. The pictures were taken by Asymetrix^®^ DVP 4.0 program (Asymetrix Corporation, Bellevue, WA, USA) in real time to observe the phase transitions in the riparin I batches.

### 3.5. Thermogravimetry (TG)

The dynamic thermogravimetric curves of bathes were recorded with a thermobalance (Shimadzu, model TGA-50) using an aluminum crucible. The apparatus was calibrated with calcium oxalate monohydrate. Experiments were conducted in the temperature range 25–900 °C, at heating rates of 10, 20 and 40 °C·min^−1^, in a flow of synthetic air and nitrogen of 20 and 50 mL·min^−1^, respectively, with a quantity of riparin I batches of exactly 5.0 ± 0.2 mg.

### 3.6. Fourier Transform Infrared (FTIR)

The FTIR spectra were recorded using a Shimadzu IRPrestige-21 spectrometer over a range of 4000–400 cm^−1^. The batches of riparin I were mixed with KBr (Vetec, Rio de Janeiro, Brazil) and compacted in a hydraulic press to obtain the KBr pellets.

### 3.7. Pyrolysis Coupled with Gas Chromatography/Mass Spectrometry (Pyr-GC/MS)

Pyrolysis studies were conducted by a pyrolyzer coupled with a gas chromatograph system (Shimadzu, GCMS-QP5050A), which interfaced directly with a mass spectrometer using electron ionization source. The fragmentation was performed by electronic impact with an ionization energy of 70 eV. The spectrometer was operated in scan mode, sweeping a mass range of *m*/*z* 50–600. The temperature of the ion source was 300 °C. A capillary column 30 m long, with 0.25 mm internal diameter and 0.25 µm particle size, was used with stationary phase phenyl:dimethylpolysiloxane (5:95). The temperature program of the column raised the temperature at a heating rate of 15 °C min^−1^ up to a final temperature of 280 °C. Helium was used as the carrier gas at a flow rate of 1.0 mL min^−1^ with a split ratio of 1:5. The samples corresponding to a powder of riparin I batches were put in a platinum crucible and introduced into the pyrolyzer at the temperatures of 250 and 500 °C for each experiment.

### 3.8. Powder X-Ray Diffractometry (PXRD)

The diffractograms of the riparin I batches were obtained using a Siemens Bruker D5000 equipment (KS Analytical Sistems, Aubrey, TX, USA) under the following operating conditions: Cu Kα radiation (*λ* = 1.5406 Å), reflection mode, with a tube voltage of 40 kV and current of 40 mA, scanning rate of 0.5° 2θ min^−1^, scanning step of 0.02° 2θ, time constant of 4 s, angular range of 5° −40° 2θ, at room temperature.

### 3.9. Solid-State ^13^C Nuclear Magnetic Resonance (ssNMR)

High-resolution ^13^C solid-state spectra for both riparin I batches were recorded using the ramp CP/MAS sequence with proton decoupling during acquisition. The solid-state NMR experiments were performed at room temperature in a Bruker Avance II spectrometer (Bruker, Karisruhe, Germany) operating at 300.13 MHz for protons and equipped with a 4-mm MAS probe. The operating frequency for carbon was 75.46 MHz. Adamantane was used as an external reference for the ^13^C spectra and to set the Harmann–Hahn matching condition in the cross-polarization experiments. The spinning rate was 10 kHz. All spectra were recorded at ambient temperature and chemical shifts were externally referenced to tetramethylsilane.

## 4. Conclusions

The reproducibility of the solid-state characteristics of riparin I was first studied in detail using various analytical techniques. This research is essential in the early stages of developing a new active pharmaceutical ingredient in order to investigate and select the characteristics of the crystalline form of interest. In the SEM images, different morphologies were observed between batches, as well as the number and agglomerates of particles on the surface of the crystals. The DSC data showed two endothermic processes in RIP-2 and RIP-3 batches, which are characteristic of crystalline forms with different melting points. The DSC data for RIP-1, RIP-4, and RIP-5 batches showed a single melting point at different temperatures and energies between batches, indicating differences in crystallinity among these batches. The TG data showed different stabilities between batches and thermal degradation in a single stage, confirmed by pyrolysis as being the volatilization of riparin I. The TG data and the FTIR spectrum did not show the presence of a solvent or water in the crystalline structure of the crystals, which may allow us to infer that these are anhydrous forms. The DSC, PXRD, and ssNMR showed different crystalline structures for RIP-1–5 batches. Due to the sensitivity of the techniques, the ssNMR spectra and pyrograms showed the presence of triethylamine in batches. This phenomenon may have resulted in changes in habits between batches. In summary, the batches of riparin I obtained by the same synthetic process had different crystal habits. The batches RIP-2 and RIP-3 showed two crystalline forms. The results highlight the need for greater control of the synthesis of this substance in order to ensure the reproducibility of the characteristics of the solid form of riparin I, batch to batch, with therapeutic efficacy and its use as an active pharmaceutical ingredient in the production of medicine.

## Figures and Tables

**Table 1:** Kinetic parameter from the dynamic TG data for riparin batches in the decomposed fraction α10 obtained using the Ozawa method.

Kinetic Parameter	Sample				
	RIP-1	RIP-2	RIP-3	RIP-4	RIP-5
Activation energy (kJ·mol^−1^)	156.85	138.45	98.87	106.50	113.45
Frequency factor	6.34 × 10^8^	5.46 × 10^8^	2.38 × 10^6^	8.23 × 10^7^	4.11 × 10^8^
Reaction order	0.0	0.0	0.2	0.0	0.4

**Table 2:** FTIR spectra recorded in KBr pellet of RIP batches 1–5.

Batches	Wavenumber (cm^−1^)			
	N–H	C=O	–C=C	
Rip-1	3325.49	1637.71	1537.27	1512.19
Rip-2	3323.56	1637.71	1539.21	1512.19
Rip-3	3323.56	1635.64	1535.34	1512.19
Rip-4	3325.49	1637.71	1539.21	1512.19
Rip-5	3325.49	1637.71	1537.27	1512.19
